# High cell density production of multimethyl-branched long-chain esters in *Escherichia coli* and determination of their physicochemical properties

**DOI:** 10.1186/s13068-016-0631-x

**Published:** 2016-10-14

**Authors:** Simón Menendez-Bravo, Julia Roulet, Martín Sabatini, Santiago Comba, Robert Dunn, Hugo Gramajo, Ana Arabolaza

**Affiliations:** 1 Microbiology Division, IBR (Instituto de Biología Molecular y Celular de Rosario), Consejo Nacional de Investigaciones Científicas y Técnicas, Facultad de Ciencias Bioquímicas y Farmacéuticas, Universidad Nacional de Rosario, Ocampo y Esmeralda (2000), Rosario, Argentina; 2 US Department of Agriculture, Agricultural Research Service, National Center for Agricultural Utilization Research, 1815 N. University St, Peoria, IL 61604 USA

**Keywords:** *Escherichia coli*, Wax production, Metabolic engineering, Fed-batch fermentation, Oleochemicals

## Abstract

**Background:**

Microbial synthesis of oleochemicals derived from native fatty acid (FA) metabolism has presented significant advances in recent years. Even so, native FA biosynthetic pathways often provide a narrow variety of usually linear hydrocarbons, thus yielding end products with limited structural diversity. To overcome this limitation, we took advantage of a polyketide synthase-based system from *Mycobacterium tuberculosis* and developed an *Escherichia coli* platform with the capacity to synthesize multimethyl-branched long-chain esters (MBE) with novel chemical structures.

**Results:**

With the aim to initiate the characterization of these novel waxy compounds, here, we describe the chassis optimization of the MBE producer *E. coli* strain for an up-scaled oil production. By carrying out systematic metabolic engineering, we improved the final titer to 138.1 ± 5.3 mg MBE L^−1^ in batch cultures. Fed-batch microbial fermentation process was also optimized achieving a maximum yield of 790.2 ± 6.9 mg MBE L^−1^ with a volumetric productivity of 15.8 ± 1.1 mg MBE (L h)^−1^. Purified MBE oil was subjected to various physicochemical analyses, including differential scanning calorimetry (DSC) and pressurized-differential scanning calorimetry (P-DSC) studies.

**Conclusions:**

The analysis of the pour point, DSC, and P-DSC data obtained showed that bacterial MBE possess improved cold flow properties than several plant oils and some chemically modified derivatives, while exhibiting high oxidation stability at elevated temperatures. These encouraging data indicate that the presence of multiple methyl branches in these novel esters, indeed, conferred favorable properties which are superior to those of linear esters.

**Electronic supplementary material:**

The online version of this article (doi:10.1186/s13068-016-0631-x) contains supplementary material, which is available to authorized users.

## Background

In the last decades, *Escherichia coli* have been highly optimized through systems metabolic engineering for the production of several bioproducts. Volatile isoprenoids [1], polyketides and non-ribosomal peptides [2], aromatic amino acids and its precursors [3], short-chain organic acids like acetate, succinate, and lactate [4], triacylglycerols (TAG) [5], and fatty acids (FA) derivatives [6, 7] constitute just a few examples of the wide variety of natural and non-natural metabolites that can be synthesized by engineered *E. coli*. In particular, the detailed biochemical and physiological understanding of its FA metabolism has determined *E. coli* as a model organism to be used as platform for high-value lipids production [8, 9]. FA derivatives, such as TAG and wax esters, can be used as a base stock for preparation of environmentally friendly biofuels and also for rapidly biodegradable lubricants [6, 9–11].

Lubricants are extensively used in industrial machineries and automobile sectors as well as for bio-medical purposes and in pharmaceutical and cosmetics industries [11–14]. The development of sustainable processes for the production of lubricants has pointed to plants oils as an alternative to the conventional mineral oil derivatives [15]. Plant oil-based lubricants, primarily constituted by TAG, show excellent lubricity and biodegradability. However, TAG shows rather poor oxidative stability and relatively high melting points. Even though chemical modifications have been reported to improve these properties, TAG still possesses relatively high oxidation rates [15–17]. On the other hand, wax esters are molecules with a high oxidation stability that already has a broad range of industrial applications in different areas, such as pharmaceutical, cosmetics, and food industries [11, 18, 19]. Jojoba oil is a liquid wax mixture extracted from the seeds of *Simmondsia californica* that is mainly composed of linear long-chain wax esters from C38 to C44 [18]. This oil has excellent physicochemical properties to be used as biolubricant and is the main natural source of commercialized wax esters [20, 21].

In 2006, Kalscheuer et al. reported the production of linear long-chain wax esters in *E. coli,* similar to those present in Jojoba oil [19]. To accomplish this goal, the authors engineered a bifunctional acyl-coenzyme A reductase from the jojoba plant and a bacterial wax ester synthase from *Acinetobacter baylyi* strain ADP1 into *E. coli*. The plant enzyme generates a long-chain alcohol from an acyl-CoA, while the bacterial wax ester synthase catalyzes the esterification of this fatty alcohol with acyl-CoA. Using this approach, wax esters, such as palmityl oleate, palmityl palmitoleate, and oleyl oleate, were produced with yields of up to 1 % of the cellular dry weight in the presence of exogenous 0.2 % (w/v) sodium oleate in the cultivation medium [19].

**Fig. 1:**
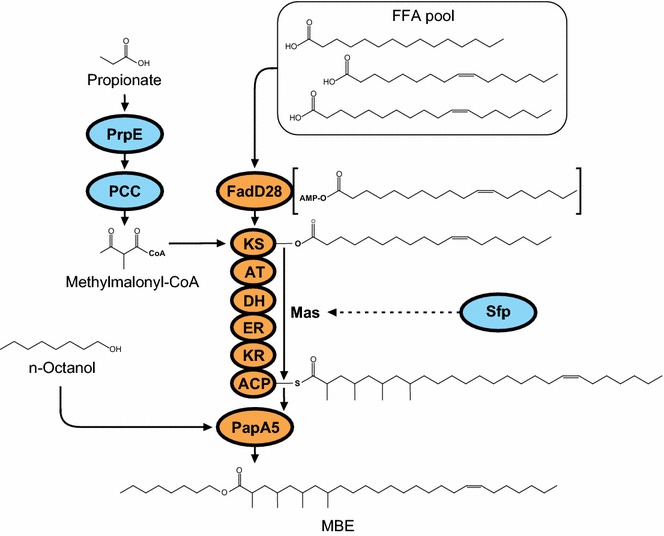
MBE biosynthesis in *E. coli* RQ1. Overexpression of native PrpE and heterologous PCC complex from *S. coelicolor* leads to the production of extender unit methylmalonyl-CoA from exogenous propionate. FadD28 allows loading of free FA (FFA) onto KS domain of Mas via its conversion into acyl-AMP derivatives. To be active, Mas must be post-translationally modified by *B. subtilis* phosphopantetheinyl transferase Sfp. Once modified, Mas is able to elongate the acyl group loaded on its KS domain using methylmalonyl-CoA as extender unit through four iterative catalytic cycles. Finally, PapA5 catalyzes a transesterification reaction between the enzyme-linked multimethyl-branched FA and exogenous *n*-octanol, giving rise to MBE. *Gray ovals* indicate protein expression from genome insertions. *White ovals* indicate protein expression from plasmids. All protein-coding genes referred in this scheme are under the control of T7 promoters

In a previous work, we used a rather different strategy to produce multimethyl-branched long-chain esters (MBE) in *E. coli* [22]. The presence of several methyl branches in these molecules is supposed to offer favorable physicochemical properties by increasing their fluidity at low temperatures, while maintaining their characteristic chemical stability [23]. For this, the mycocerosic acid biosynthetic pathway from *Mycobacterium tuberculosis* was engineered into a methylmalonyl-CoA producer *E. coli* strain. This pathway is constituted by the mycocerosic acid synthase Mas, which is an iterative type I polyketide synthase (PKS), the fatty-acyl-AMP ligase FadD28, and the polyketide-associated protein PapA5 [24]. FadD28 is required for the activation of FA into their acyl-AMP derivatives and their loading on the KS domain of Mas. This PKS elongates a C16–C18 FA derivative using methylmalonyl-CoA as a substrate, generally by four catalytic cycles, and the tetramethyl-branched FA is finally transferred to a phthiocerol molecule by the PapA5 acyltransferase. The final product is phthiocerol di-mycoserosate (PDIM) [24]. Considering that in vitro PapA5 could utilize other alcohols as acceptors [25], we coupled these three enzymes with the endogenous FA synthetic machinery of *E. coli* RQ1 (Table 3) [22]. By feeding propionate and *n*-octanol to the growth medium, *E. coli* RQ1 expressing Mas, FadD28, and PapA5 was able to synthesize MBE (Fig. 1) [22]. Furthermore, due to the high flexibility of this heterologous biosynthetic pathway towards the FA and the alcohol moieties, this *E. coli* platform allowed the synthesis of a wide set of related molecules by feeding different alcohols and different long-chain FA to the culture medium [22].

In this work, we focused on the physicochemical characterization of the MBE produced by *E. coli* RQ1. Considering that one of the most challenging issues about producing high-value lipids in engineered microbes is the poor productivity achieved, we aimed to optimize both the bacterial chassis and the MBE production bioprocess. Therefore, here, we describe the improvement of strain RQ1, the design of an optimized fed-batch fermentation process, its scaling up to 75 L, and the development of an extraction method for MBE purification. Finally, we report physicochemical data corresponding to low-temperature behavior and thermal stability of the purified MBE oil.

## Results and discussion

### Metabolic engineering to improve MBE production

In a previous work, a rational design for the optimization of the MBE producer platform was initiated [22]. For this, strain RQ1 was transformed with an integrative plasmid containing ´*tesA*, a leaderless version of the *E. coli* periplasmic thioesterase [26]. The derivative strain was named RQ2. This strain produced 97.9 ± 5.2 mg MBE L^−1^ in bioconversion assays carried out in batch cultures, a 28.6 % increase compared with RQ1 [22]. This was the highest titer reported for MBE production. However, the final cell density for this strain was slightly reduced compared with the parental strain grown under the same conditions. This phenotype became evident during fed-batch cultures: RQ2 reached a final OD_600_ of 50 ± 4.3 after 30 h of fermentation, while RQ1 exhibited a final OD_600_ of 110 ± 7.2 after 30 h. The cause of this growth impairment is likely to be the toxicity generated by the high free FA levels reached as a result of the deregulation in lipid metabolism caused by the expression of ‘TesA. Therefore, with the final aim of establishing a complete scaled-up bioprocess, we performed different engineering strategies to optimize MBE titers in RQ1. These strategies were focused on the improvement of two main metabolic aspects that govern MBE biosynthesis: (i) the expression levels of the biosynthetic genes and (ii) the pools of substrates. It is worth to note that bioconversion assays performed to evaluate the different strategies were carried out under exogenous *n*-octanol supplementation. This was done, because biochemical characterization of PapA5 indicated that *n*-octanol is the preferred substrate for this enzyme in vitro [25]. This evidence, together with our previous results [22], suggested that *n*-octanol supplementation is the more convenient culture condition to obtain high MBE titers in fed-batch fermentation.

**Fig. 2:**
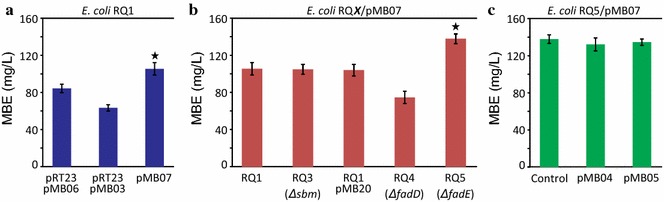
Optimization of MBE production. *E. coli* RQ1 derivative strains harboring indicated plasmid(s) were grown in LB and, when culture reached OD_600_ ~ 1 induction was carried out by the addition of 0.5-mM IPTG and 0.2 % l-Ara. Immediately after induction, cultures were supplemented with 20-mM propionate, 2-mM oleate, and 1-mM *n*-octanol, and were incubated at 22 °C for 24 h. After that, cells were harvested and total lipid were extracted and further processed as described in “Methods”. **a** MBE accumulation in response to variations in the gene expression system. **b** MBE production in RQ1/pMB07 derivatives strains where different genes were knocked out. As it is mentioned in the main text and described in “Methods”: RQ3 = RQ1 Δ*sbm*; pMB20 = pCA30:T7-*accA2*-*pccE*-*pccB*; RQ4 = RQ1 Δ*fadD*; RQ5 = RQ1 Δ*fadE*. **c** MBE titers for RQ5/pMB07 cells containing indicated plasmids and expressing individual extra copies of *papA5* (pMB04) or *fadD28* (pMB05). *Error bars* represent the SD of three experiments conducted by triplicate. *Stars* mean significant differences respect to empty plasmid control strain (*p* < 0.05)

We initiated the MBE pathway optimization process by examining the use of alternative promoters, genes organization, and plasmid copy numbers to modulate the expression levels of the basic MA-system genes—*fadD28*, *mas,* and *papA5*—in RQ1. For this, we constructed different expression vectors derived from plasmids pET28a or pBAD33 harboring different combinations of the MA-system genes as independent transcription units or as part of designed operons (Table 3). The analysis of MBE production in each derivative strain containing different combinations of the three genes indicated that *papA5, fadD28,* and *mas,* assembled as a single T7 controlled operon into a pET28 derivative vector (pMB07), resulted in the highest yields (Fig. 2a). For instance, RQ1/pMB07 produced 105.7 ± 6.7 mg MBE L^−1^, while RQ1/pRT23/pMB06 produced 84.6 ± 4.5 mg MBE L^−1^and RQ1/pRT23/pMB03 produced 63.6 ± 3.4 mg MBE L^−1^ under the same culture conditions (Fig. 2a). Therefore, RQ1/pMB07 was utilized for further studies.

For supplying (2*S*)-methylmalonyl-CoA substrate, RQ1 carries an artificial operon containing the *accA2*-*pccE*-*pccB* genes coding for the propionyl-CoA carboxylase (PCC) complex of *S. coelicolor* under the T7 promoter inserted into the *ygfG*-*ygfH* genes. Besides YgfG and YgfH, Sbm is also involved in the propionate metabolism of *E. coli;* this protein catalyzes the conversion of (2R)-methylmalonyl-CoA to succinyl-CoA [27]. Therefore, we decided to further delete the *sbm* gene in our producer strain RQ1. Since there is no solid evidence to dismiss the existence of an *E. coli* epimerase that could convert (2S)-methylmalonyl-CoA to (2R)-methylmalonyl-CoA, the objective of the *sbm* deletion was to avoid the drain of (2S)-methylmalonyl-CoA to succinyl-CoA. Deletion of *sbm* in RQ1 yielded strain RQ3. This strain was first characterized under conditions meant to maximize MBE levels, namely, expression of *S. coelicolor* PCC genes and optimal addition of exogenous propionate, *n*-octanol, and oleate (see “Methods”). However, our results indicate that deletion of *sbm* did not impact MBE production; titers for RQ3/pMB07 did not show significant differences compared with those of RQ1/pMB07 (Fig. 2b). These studies suggest that either the Sbm pathway does not affect the availability of (2S)-methylmalonyl-CoA, or that the levels of this metabolite are not limiting MBE production in this genetic background. To get further insights into this issue, we applied another strategy towards the improvement methylmalonyl-CoA levels. The hypothesis was that a second copy of the PCC complex coding genes could increase the levels of (2S)-methylmalonyl-CoA. Thus, to assess this possibility, the *accA2*-*pccE*-*pccB* designed operon was cloned into the pCA30 vector—which contains the p15A origin of replication and a T7 promoter sequence—yielding pMB20, and then introduced into RQ1/pMB07. Although RQ1/pMB07/pMB20 showed increased levels of PCC proteins, as determined by Western Blot assays (Additional file 1: Figure S1A), there was no increase in MBE production compared with RQ1/pMB07 (Fig. 2b). In summary, all these results strongly suggest that the levels of (2S)-methylmalonyl-CoA are not a limiting factor or a bottleneck for MBE biosynthesis. Therefore, in a next step, we sought to increase the levels of free FA, a substrate of the fatty-acyl-AMP ligase FadD28. To accomplish it, we proceeded to block the FA β-oxidation pathway by disrupting *fadE* and *fadD* genes. FadD activates free FA into the metabolically active acyl-CoA thioesters [22], and FadE is an acyl-CoA dehydrogenase that catalyzes the oxidation of an acyl-CoA to a 2-enoyl-CoA derivative [23]. Disruption of *fadD* in RQ1 yielded strain RQ4; and disruption of *fadE* in RQ1 yielded strain RQ5. RQ4/pMB07 did not show improved MBE levels compared with RQ1/pMB07 (Fig. 2b). On the other hand, RQ5/pMB07 exhibited a 30.6 % increase in MBE production when compared with RQ1/pMB07 (Fig. 2b). That is, RQ5/pMB07 produced 138.1 ± 5.3-mg MBE L^−1^, whereas RQ1/pMB07 produced 105.7 ± 6.7-mg MBE L^−1^ under the same culture conditions (Fig. 2b). All the recombinant strains displayed the same growth kinetics and comparable final OD_600_. Accordingly, RQ5/pMB07 was selected as the best MBE-producing strain to continue with our studies.

It is widely reported that the expression of heterologous genes from inducible promoters can lead to imbalances of the heterologous proteins, resulting in suboptimal product titers [28–32]. Considering that the Mas enzyme represents the most abundant protein in our MBE-producing system (Additional file 1: Figure S1B), we performed gene dosage experiments to pinpoint whether PapA5 or FadD28 enzymes were the limiting factor of MBE production in RQ5/pMB07. For this, *papA5* and *fadD28* genes were individually subcloned into the p15A-based expression vector pBAD33 (yielding pMB04 and pMB05, respectively) and introduced into RQ5/pMB07. We observed that, even though PapA5 and FadD28 protein levels were higher in each derivative strain (Additional file 1: Figure S1C, D), none of these modifications resulted in improved MBE titers (Fig. 2c). Several publications have reported that a high metabolic burden may be imposed to the cell when two plasmids carrying heterologous proteins coding genes are introduced in the same system [9, 33]. However, no phenotypic differences were observed between RQ5/pMB07, RQ5/pMB07/pMB04, and RQ5/pMB07/pMB05, as they all showed the same growth behavior (data not shown). Considering this, it seems clear that neither PapA5 nor FadD28 is titer-limiting factors for MBE production. Overall, these results might suggest that other more general metabolic constrains, like redox imbalance, energetics impairments, or product toxicity, among others, are restricting the capability of the cell to produce higher levels of MBE.

**Fig. 3:**
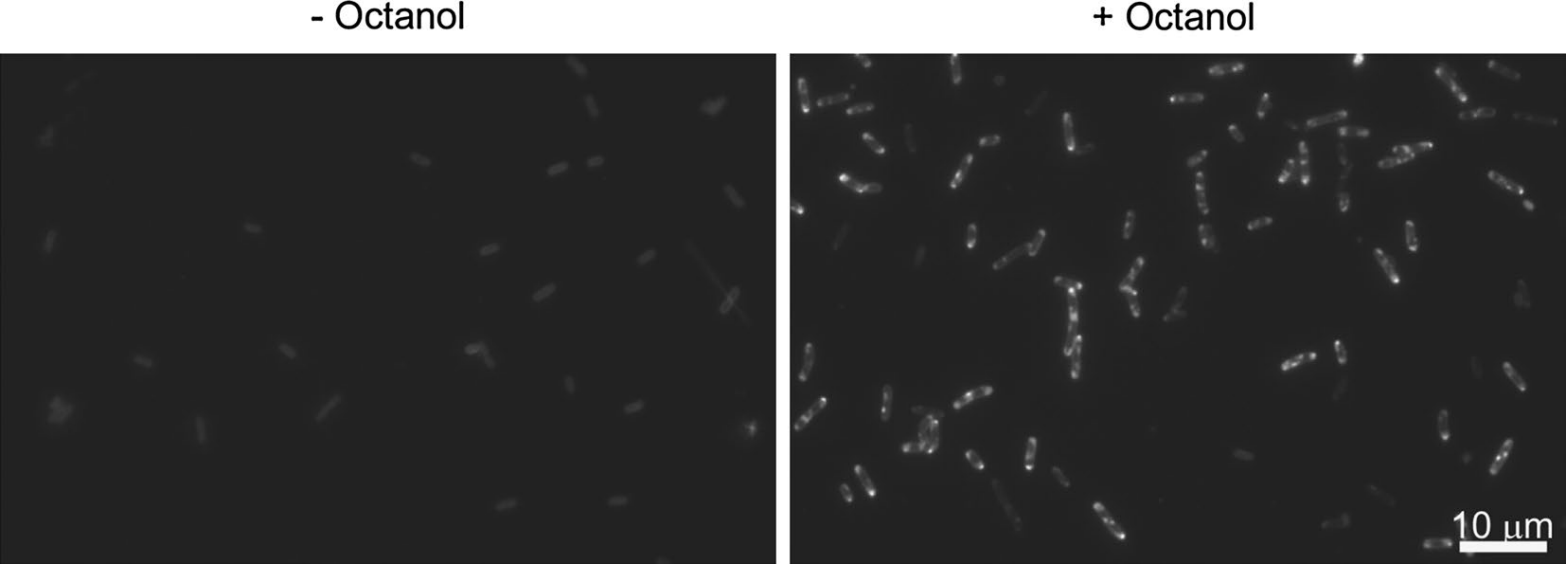
Fluorescence micrographs of Nile Red stained *E. coli* RQ5/pMB07 cells. Cultures grown in LB were induced by the addition of 0.5-mM IPTG and supplemented with 20-mM propionate when OD_600_ ~ 1 was reached. Immediately after, cultures were split into two and just one of the halves was supplemented with 1 mM *n*-octanol. At different time points, samples were taken and processed as described in “Methods”. Shown micrographs were taken 8 h after induction. The absence of *n*-octanol in the culture media leads to no MBE accumulation (*left*), whereas addition of *n*-octanol derives in MBE production and appearance of intracellular fluorescent bodies 8 h after induction (*right*)

Microscopic examination of Nile Red stained RQ5/pMB07 cells revealed the presence of fluorescent bodies 8 h after the addition of 0.5-mM IPTG (Fig. 3), suggesting that that MBE are able to cluster together, giving rise to intracellular neutral lipid inclusions. Accordingly, Elbahloul and Steinbüchel [34] observed a similar phenotype for FA ethyl ester-producing cells of *E. coli* harboring p(Microdiesel) vector [34]. This technique was considered as a rapid screening to determine the minimal after induction time lapse needed for the initiation of MBE biosynthesis.

In summary, the studies carried out towards optimizing MBE production led us to identify RQ5/pMB07 as the most suitable strain to carry out a high cell density MBE-producing fed-batch bioprocess.

### Development of a fed-batch fermentation process for MBE production

To produce MBE at a pilot scale level, a high cell density fed-batch bioprocess was developed. To set the cultivation conditions, 1 L fed-batch fermentations were carried out using strain RQ5/pMB07. Variables evaluated were: (1) length of batch phase; (2) OD_600_ at which IPTG induction was carried out; (3) IPTG concentration; (4) post-induction temperature; (5) concentration and time point(s) at which precursors were supplied; and (6) total duration of cultivation. Since MBE are intracellularly stored (Fig. 3), both final cell density and amount of MBE produced per DCW were considered parameters to optimize. Therefore, and considering that MBE yields obtained with glucose as carbon source are 1.5-fold higher than those obtained with glycerol (Additional file 2: Figure S2), glucose was used as sole carbon source. Cultures were grown in a modified version of the defined medium described in Lau et al. [35] (see “Methods”). Overall, five different cultivation conditions were tested and three independent fermentations for each condition were carried out.

**Fig. 4:**
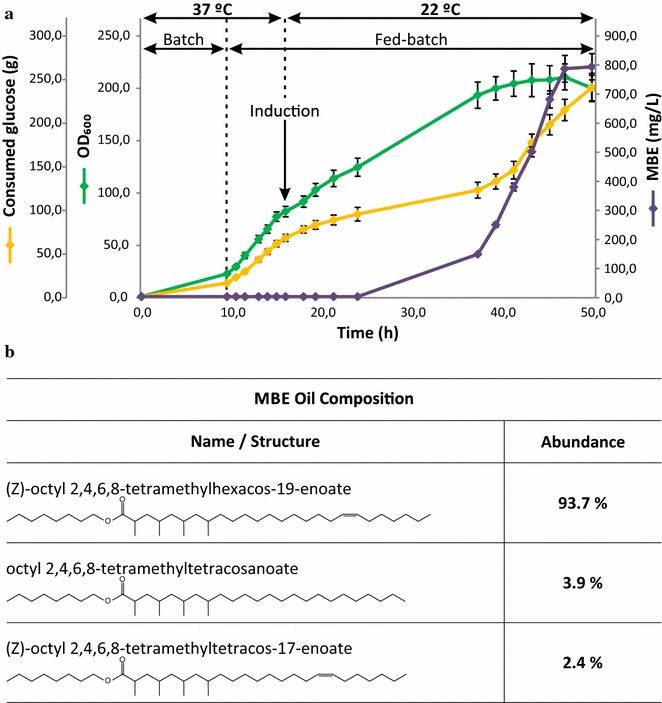
Time-course diagram of fed-batch fermentation of *E. coli* RQ5/pMB07 performed under cultivations conditions #3 and composition of derived MBE oil. **a** RQ5/pMB07 was cultured as described in “Methods”, Table 1 (cultivation conditions #3) and main text. Samples were taken periodically for OD_600_, glucose, and neutral lipids analysis. *Error bars* represent the SD of three independent experiments performed under the same cultivation conditions. **b** Composition of the MBE oil obtained under cultivation conditions #3 as determined by LC–MS analysis

Fermentations carried out under cultivation condition #1 exhibited a MBE productivity of 13.7 ± 0.9 mg (L h)^−1^ (Table 1). The complete cultivation process lasted 30 h with an initial 6.0 ± 0.4-h batch phase. The duration of the batch phase was entirely determined by the initial glucose concentration (in this case, 5 g L^−1^). Induction was carried out by the addition of 0.5 mM IPTG at OD_600_ 68.1 ± 5.1 (12.5 ± 0.9-h cultivation time). At this point, 40-mM propionate, 2-mM *n*-octanol, and 0.07-mM oleic acid were added to the culture, and temperature was lowered to 22 °C. Eight hours after IPTG induction, MBE biosynthesis became detectable. This was in agreement with the results obtained for the same strain in batch cultures. Twelve hours after induction, another pulse of 0.07-mM oleic acid was added to the culture. Final OD_600_ was 114.3 ± 8.1 and an OD_600_ peak of 124.1 ± 8.9 was registered at approximately 28 h cultivation time. Since the culture exhibited cellular lysis after an OD_600_ of approximately 124, we aimed to extend the time period comprehended between the induction and the time at which the highest OD_600_ is reached. Therefore, in fermentations carried out under cultivation conditions #2, IPTG induction was carried out at a lower OD_600_ and the fed rate of glucose after induction was reduced. Precursors supply and post-induction temperature were maintained in the same setup as in cultivation condition #1. Hence, induction was carried out at OD_600_ 45.0 ± 2.8 and the total duration of the fermentation was 50-h (post-induction phase of approximately 40 h). Under these conditions, final OD_600_ was 54.3 ± 2.9 and the maximum OD_600_ reached was 74.0 ± 4.1. This result suggested that the maximum OD_600_ is determined mainly by the OD_600_ at which induction is performed. Indeed, reproducibility between replicates indicated that, after induction, cultures go through approximately one round of duplication before cellular lysis become evident. This post-induction behavior seems to be independent of the rate at which culture is growing. Therefore, further modifications were oriented towards the maximization of the OD_600_ at which induction was carried out. In the fermentations performed under cultivation conditions #3, the batch phase was extended to take maximum advantage of the time period at which culture is growing at its highest growth rate (Table 1; Fig. 4). To accomplish that, the initial glucose concentration was set to 20 g L^−1^. Fed-batch phase was initiated at OD_600_ 22.1 ± 1.2 and induction was carried out at OD_600_ 81.3 ± 4.9. IPTG concentration was reduced to 0.1 mM to reduce the metabolic impact of heterologous proteins expression. Oleic acid supply was carried out in the same way as in conditions 1 and 2. Propionate and *n*-octanol supply were performed in three pulses of 20 mM and 1 mM each, respectively, separated by 12 h to minimize cellular damage due to solvent toxicity. Total duration was 50 h and final OD_600_ was 199.2 ± 12.1, with a maximum OD_600_ of 209.8 ± 12.4 registered at approximately 47 h. Final titer reached under this conditions was 790.2 ± 6.9-mg MBE L^−1^ (Table 1; Fig. 4), which corresponds to a productivity of 15.8 ± 1.1 mg MBE (L h)^−1^. Further modifications in the fermentation conditions were tested to evaluate the effect of post-induction temperature and inducer concentration on MBE production. Fermentations carried out under cultivation condition #4 were started with the same initial glucose concentration as in cultivation condition #3. Induction and precursors supply were also performed in the same way. However, in this case, temperature was lowered to 30 °C after induction. The objective of this modification was to shorten the time period needed to achieve one doubling round after induction to reduce the total duration of the process and, therefore, fermentation costs. Nevertheless, the maximum DO_600_ reached was 142.1 ± 13.9 and MBE productivity was the lowest of the five cultivation conditions tested (5.3 ± 0.6 mg MBE (L h)^−1^). This result suggests that protein expression must be suboptimal at 30 °C and that a metabolic burden triggered by that may affect cellular viability. Therefore, in fermentations performed under the cultivation condition #5, IPTG concentration was reduced to 0.05 mM and temperature after induction was set to 25 °C. Although the productivity obtained under these conditions was high, it remained lower than the one obtained under cultivation condition #3 (10.9 ± 0.8 mg MBE (L h)^−1^ vs 15.8 ± 1.1 mg MBE (L h)^−1^, respectively). Fermentations carried out under cultivation conditions #4 and #5 indicated that post-induction temperature is critical to both MBE production and biomass accumulation. Based on these studies, we defined protocol #3 as the best fed-batch conditions for MBE production when using *E. coli* RQ5/pMB07 as a platform (Table 1; Fig. 4). Accordingly, this fermentation protocol was applied to a 75-L pilot-plant with the objective of producing enough MBE oil to determine physicochemical properties related with its thermal behavior.

**Table 1:** Cultivation of *E. coli* RQ5/pMB07 under different fed-batch fermentations conditions

Parameter	Cultivation Conditions				
	#1	#2	#3	#4	#5
Total duration (h)	30	50	50	40	40
Batch phase duration (h)	6.0 ± 0.4	6.2 ± 0.3	9.5 ± 0.6	10.1 ± 0.9	10.4 ± 0.9
Initial glucose conc. (g L^−1^)	5	5	20	20	20
OD_600_ at which fed-batch phase started	8.1 ± 0.6	8.0 ± 0.4	22.1 ± 1.2	23.2 ± 2.6	23.7 ± 2.1
Induction time point (h)	12.5 ± 0.9	10.5 ± 0.7	16.2 ± 1.1	15.9 ± 1.4	15.3 ± 1.5
OD_600_ of induction	68.1 ± 5.1	45.0 ± 2.8	81.3 ± 4.9	79.6 ± 7.3	80.4 ± 7.0
IPTG conc. (mM)	0.5	0.5	0.1	0.1	0.05
Propionate conc. (mM)	40	40^b^	60^b^	60^b^	60^b^
n-Octanol conc. (mM)	2	2^c^	3^c^	3^c^	3^c^
Oleic acid supply (mM)	0.14^a^	0.21^a^	0.21^a^	0.21^a^	0.21^a^
Post-induction growth rate (h^−1^)	0.04	0.02	0.04	0.04	0.04
Post-induction temperature (°C)	22	22	22	30	25
Maximum OD_600_	124.1 ± 8.9	74.0 ± 4.1	209.8 ± 12.4	142.1 ± 13.9	121.3 ± 11.0
Final OD_600_	114.3 ± 8.1	54.3 ± 2.9	199.2 ± 12.1	122.8 ± 11.1	97.0 ± 8.9
Productivity (mg MBE (L h)^−1^)	13.7 ± 0.9	5.9 ± 0.3	15.8 ± 1.1	5.3 ± 0.6	10.9 ± 0.8
Yield of MBE (mg MBE (g glucose)^−1^)	2.61 ± 0.1	2.13 ± 0.1	2.09 ± 0.2	0.98 ± 0.1	2.51 ± 0.3
Final Titer (mg MBE (L)^−1^)	411.5 ± 5.5	295.3 ± 3.7	790.2 ± 6.9	212.1 ± 1.8	436.8 ± 3.9

Batch phase duration, OD600 values and productivities are expressed as media ± SD of three independent fermentations carried out for each condition

^a^Total quantity added in pulses of 0.07-mM oleic acid every 12-h post-induction

^b^Total quantity added in pulses of 20-mM propionate every 12-h post-induction

^c^Total quantity added in pulses of 1-mM *n*-octanol every 12-h post-induction

### MBE thermo-oxidative stability and low-temperature properties

The physical and chemical properties of plant oils are mostly determined by their FA composition. Unsaturated bonds in the acyl groups increase oil fluidity, which leads to favorable cold temperatures behavior; however, they also increase their rate of oxidation, which results in poor thermal stability [16, 17, 21, 30]. On the other hand, saturated long-chain molecules are chemically stable, although they have high melting points which usually results in poor low‐temperature flow characteristics [10]. Different modern technological approaches have been developed to solve the problems associated with the application of plant oils in biolubricants. For example, modification of unsaturated FA by attaching functional groups at the sites of unsaturation has demonstrated a high potential for improving some properties for their use as biolubricant basestocks [10]. In this context, we used a completely different approach to obtain branched‐chain FA derivatives and we hypothesized that the bacterial produced MBE presents favorable low‐temperature performance while maintaining optimal thermo‐oxidative characteristics.

**Fig. 5:**
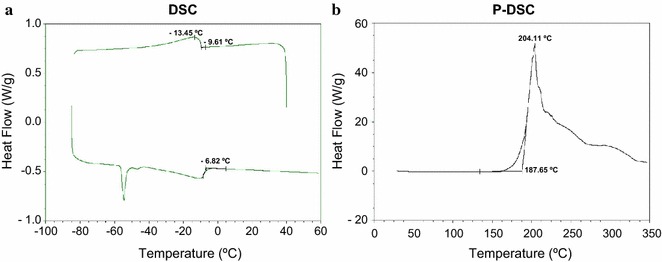
Differential scanning calorimetry (DSC). DSC measures changes in heat flowing through the sample as the oil is heated or cooled between solid and liquid phases. P-DSC measures changes in heat flowing through the sample as it is heated under a constant flow of oxygen as purge gas. **a** Cooling (*top*) and melting (*bottom*) transitions of the MBE measured by DSC. Transitions were characterized by the onset temperatures annotated next the cooling (−9.61 °C) and heating (−6.82 °C) scans in the figure. Refer to Table 3 for transition temperatures. **b** Onset temperature (187.65 °C) obtained for the onset of thermo-oxidative degradation measured by P-DSC for the MBE sample. See “Methods” for technique details

Differential scanning calorimetry (DSC) is an accepted technique for the thermal characterization of oils and fats [36, 37]. The range of low‐temperature performance and the oxidation stability of MBE oil were measured using DSC and pressurized‐DSC (P-DSC), respectively. Representative DSC and P-DSC curves obtained for MBE sample are reported in Fig. 5. Table 2 shows the results obtained for MBE and summarizes different parameters reported in literature for jojoba oil and its main constituent’s esters, as well as for epoxidized ricinoleic acid and epoxidized oleic acid [15, 20, 36, 38, 39]. Comparing with jojoba oil, a mix of waxes mainly composed of C20:1 FA and C20:1 and C22:1 fatty alcohols [20, 38], the MBE produced by RQ5 exhibits a lower pour point (PP). When compared with synthetic jojoba oil-like esters [38], MBE exhibits lower values of crystallization onset temperature (T_O_) and completion of melt temperature (T_COM_) (Table 2). These results indicate that MBE are likely to possess cold flow properties superior to those of a widely used industrial wax-based extract like jojoba oil. Regarding chemical stability, the MBE sample exhibits very high values of oxidation onset temperature (T_OX_) and signal maximum temperature (T_PK_) (Table 2), being the last one the temperature at which maximum heat output is observed in the sample during oxidative degradation. Considering T_OX_ values reported in literature for epoxidized ricinoleic and oleic acids [15, 39], it can be observed that MBE constitutes a thermally stable material.

**Table 2:** Physicochemical properties of MBE regarding thermal behavior

Material		PP (°C)	DSC		P-DSC	
			T_O_ (°C)	T_COM_ (°C)	T_OX_ (°C)	T_PK_ (°C)
A	MBE	–6.00	–9.36	–6.27	188.18	204.03
	Methyl oleate	–	–42.50	–17.66	164.00	ND
	Methyl tetracosanoate	58.60	55.33	60.07	ND	ND
B	Jojoba oil [20, 38, 40]	9.00	12–15^a^	16–25^a^	188	208
	Epoxidized ricinoleic acid [15]	9.00	–	–	60	228
	Epoxidized oleic acid [39]	0.00	–	–	75	164

*ND* not determined

*A* values obtained from PP, DSC, and P-DSC analyses of MBE wax esters as well as methyl oleate and methyl tetracosanoate used as references (see “Methods”). Data shown represent the mean of independent experiments, SD ≤ 0.59

*B* values obtained from the literature for jojoba oil, jojoba oil synthetic esters and epoxidized ricinoleic and oleic acids

^a^Values corresponding to C40 jojoba wax-like synthetic esters [38]

Considering the chemical structures of MBE and that corresponding to the esters that mainly confirm jojoba oil, it can be seen that the principal difference between them is the presence of methyl branches in MBE. Considering this, the properties measured would validate our initial hypothesis in terms that it is likely that these methyl branches account for the better cold temperature properties of MBE and, at the same time, do not interfere with their thermo-chemical stability. By possessing improved values than those reported for jojoba oil, we are enabled to assume that MBE would have an expanded plethora of industrial applications.

## Conclusions

In this work, we genetically engineered our *E. coli* MBE producer strain making an improvement of 30.6 % in the final titer in batch cultures. After that, a fed-batch microbial fermentation process was designed and optimized, achieving a maximum yield of 790.2 ± 6.9-mg MBE L^−1^ and a volumetric productivity of 15.8 ± 1.1-mg MBE (L h)^−1^. Under these cultivation conditions, addition of propionate, *n*-octanol, and oleic acid to growth medium was carried out. Although addition of exogenous substrates is not convenient from an economically point of view, this was done to obtain maximum yield of MBE. Finally, we scaled-up the production of bacterial MBE, developed a purification method, and measured physicochemical properties related to their thermal behavior.

DSC T_O_ and T_COM_ values, as well as PP, revealed that these molecules exhibit a more extended range of work at low temperatures than jojoba oil, that is, a better fluidity and enhanced cold‐temperatures performance. At the same time, MBE present T_OX_ and T_PK_ values close to those corresponding to jojoba oil, indicating that the thermo-oxidative properties of both oils are comparable.

These results encourage us to further characterize MBE oil by extensive physicochemical properties determinations, such as measurement of viscosity index and refraction coefficient. This will give us a more precise idea about concrete MBE applications, as well as generating a more clear comprehension of the principles that underlie the relationship between chemical structure and possible industrial uses for oleochemicals. Finally, given the remarkable diversity of MBE compounds that could be synthesized in vivo, it is likely that a similar microbial engineering approach can yield high quality or “selected” esters of designed structures suitable for defined or specific applications.

## Methods

### Media and growth conditions

*Escherichia coli* strains were grown either on solid or in liquid Luria–Bertani medium (LB; 10-g Bacto Tryptone, 5-g yeast extract, and 10-g NaCl per liter) at 37 °C and supplemented when needed with the following antibiotics: 100 μg mL^−1^ ampicillin (Ap), 50-μg mL^−1^ kanamycin (Km), and 20 μg mL^−1^ chloramphenicol (Cm).

### Strain construction

Strains RQ3 and RQ4 were constructed by the one-step inactivation of *E. coli* chromosomal genes described by Datsenko and Wanner [41]. For this, sbm_F/sbm_R and fadD_F/fadD_R oligonucleotides were used to amplify the Km cassette of pKD13 plasmid. The resulting DNA fragments were used to transform RQ1/pKD46 electrocompetent cells to replace *sbm* and *fadD* genes, respectively. The kanamycin resistance cassette was deleted by FLP recombinase-mediated excision, yielding strains RQ3 and RQ4 [41]. Strain RQ5 was constructed by P1 transduction of *fadE*::*Km* marker from strain JW5020 [42] to RQ1 and subsequent FLP recombinase-mediated excision of kanamycin cassette [41]. Deletions of *sbm, fadD* and *fadE* were checked by PCR using the primers, sbm_check_F/R, fadD_check_F/R, and fadE_check F/R.

### Plasmid construction

All the oligonucleotide primers and plasmids used in this work are listed in Table 3 and all the heterologous enzymes expressed in RQ strains are detailed in Table 4. To express genes from the T7 promoter in a pET28a compatible plasmid, pCA30 vector was constructed as following described: First, the p15A origin of replication and Cm^R^ cassette sequences from pBAD33 vector were amplified by PCR using the SB122_F/R primer pair. After purification of the DNA fragment, it was digested with *Xba*I and self-ligated to yield pSB122 plasmid. T7 promoter sequence was PCR-amplified from pET28a vector using SB123_F/R primer pair. The resulting DNA fragment, named SB123, was subsequently cloned into pCR^®^-BluntII-TOPO vector and checked by DNA sequencing (Maine University DNA sequencing facility, USA). After that, the SB123 sequence was isolated as an *Xba*I/*Pst*I digestion fragment and cloned into *Xba*I/*Pst*I restriction sites of plasmid pSB122, yielding the pCA30 expression vector.

**Table 3:** Primers, plasmids, and strains used in this work

Primer name	Sequence	Reference
SB122_F	CTCTAGATGAATTCTATATCGCCGACATCACC	This work
SB122_R	CTCTAGAAGCTGCAGGCGTTTAAGGGCACCAATAA	This work
SB123_F	GAATTCGCTTCTAGAGCGATATAGGCGCCAGCAAC	This work
SB123_R	CTGCAGAGCTACTAGTGGGGAATTGTTATC	This work
fadD_F	CATTTGGGGTTGCGATGACGACGAACACGCATTTTAGAGGTGAAGAATTGTGTAGGCTGGAGCTGCTTCG	This work
fadD_R	TAACCGGCGTCTGACGACTGACTTAACGCTCAGGCTTTATTGTCCACTTTATTCCGGGGATCCGTCGACC	This work
sbm_F	CGTAGGCGCAAATACCCTCATTTTGATTGCGTTTTACGGAGCAAATAATGATTCCGGGGATCCGTCGACC	This work
sbm_R	GGCGAATACTTTCTGCCAGCGTGGCTTCATTAATCATGATGCTGGCTTATTGTAGGCTGGAGCTGCTTCG	This work
fadE_check_F	CAAAAGCGAGAAGTACGGGCAGGTG	This work
fadE_check_R	CACCGTTTTACCATCAATCGAAAGC	This work
fadD_check_F	GTATAATCCCGGCCCCGCGAGAGTA	This work
fadD_check_R	ATGGAGAAATTTTGTGATGGACGGA	This work
sbm_check_F	GGTCACAAAGTCCTTCGTCAG	This work
sbm_check_R	GTCATTCATGCGGGTTTTATC	This work

Plasmid	Description	Reference
pET28a	Vector for expression of N-terminal His-tagged proteins under the T7 promoter; Km^R^	Novagen
pKD13	Template plasmid for amplification of the FRT-flanked kanamycin cassette; Ap^R^ Km^R^	[41]
pKD46	Temperature-sensitive replication plasmid for Red recombinase expression; Ap^R^	[41]
pCP20	Temperature-sensitive replication plasmid for thermal induction of FLP synthesis; Cm^R^ Ap^R^	[43]
pCR^®^-BluntII-TOPO	Vector used for cloning of blunt PCR products; Km^R^	Invitrogen
pBAD33	Vector for protein expression under P_BAD_ promoter; Cm^R^	[44]
pCC05	pET28a derivative vector containing *accA2*, *pccE* and *pccB* genes; Km^R^	[22]
pRT23	pET21 derivative vector containing *mas* gen; Ap^R^	[24]
pMB03	pET28 derivative vector containing *papA5* and *fadD28*; Km^R^	[22]
pMB04	pBAD33 derivative vector containing *papA5*; Cm^R^	[22]
pMB05	pBAD33 derivative vector containing *fadD28*; Cm^R^	[22]
pMB06	pBAD33 derivative vector containing *papA5* and *fadD28*; Cm^R^	[22]
pMB07	pET28 derivative vector containing *papA5*, *fadD28* and *mas*; Km^R^	[22]
pSB122	Vector containing the p15A origin of replication from pBAD33; Cm^R^	This work
pCA30	pSB122 derivative vector containing T7 promoter; Cm^R^	This work
pMB20	pCA30 derivative vector containing *accA2, pccE and pccB* genes; Cm^R^	This work
pMB21	pCA30 derivative vector containing *E. coli fadR* gene; Cm^R^	This work

Strain	Description	Reference
DH5α	*E. coli* K12 F^−^ *lacU169 (Φ80lacZΔM15) endA1 recA1 hsdR17 deoR supE44 thi*-*1 l2 gyrA96 relA1*	[45]
BAP1	*E. coli* F^−^ *ompT* *hsdSB (rB*-*mB*-*) gal dcm (DE3) prpRBCD:: T7prom*-*sfp*-*T7prom*-*prpE*	[46]
RQ1	BAP1 *ygfGH::T7prom*-*accA2*-*pccE*-*pccB*-*T7term*	[22]
RQ3	*RQ1 Δsbm*	This work
RQ4	*RQ1 ΔfadD*	This work
RQ5	*RQ1 ΔfadE*	This work

**Table 4:** Enzymes used in this stud

Gene and enzyme description	Organism	Locus tag	Accession number
*fadD28*, acyl-AMP ligase	*M. tuberculosis H37Rv*	Rv2941	I6XFQ7
*mas*, mycocerosic acid synthase	*M. tuberculosis H37Rv*	Rv2940c	I6Y231
*papA5*, acyl-ACP transesterase	*M. tuberculosis H37Rv*	Rv2939	I6YAN2
*accA2,* acyl-CoA carboxylase α subunit	*S. coelicolor* A3 (2)	SCO4921	Q9X4K7
*pccB,* propionyl-CoA carboxylase β subunit	*S. coelicolor* A3 (2)	SCO4926	Q9EWV4
*pccE*, propionyl-CoA carboxylase ε subunit	*S. coelicolor* A3 (2)	SCO4925	Q9EWV8
*sfp*, phosphopantetheinyl transferase	*B. subtilis*	BSU03570	X63158
*prpE*, propionyl-CoA synthetase	*E. coli*	b0335	P77495

pMB20 was constructed by ligation of the *Xba*I/*Spe*I digest of pCC05 into pCA30 *Spe*I site. Correct orientation of the insert was then checked with restriction enzymes.

### Bioconversion assay

Overnight cultures of the corresponding strains were used to inoculate fresh LB medium supplemented with 20-μM biotin and the appropriate antibiotics. At mid-exponential phase, cultures were induced with 0.5-mM IPTG and supplemented with 1-mM octanol, 30-mM sodium propionate, and 2-mM oleate + 0.4 % (w/v) Brij-58, when needed. The induced cultures were further incubated for 20 h at 22 °C. Cells were harvested and total lipids were extracted from supernatant and cell material according to the Bligh and Dyer method [47]. Total lipid extracts were analyzed by TLC on silica gel 60 F254 plates (0 ± 2 mm, Merck) using the solvent system hexane/diethylether/acetic acid (90:7.5:1, v/v/v). Lipids were visualized by Cu-phosphoric staining.

### Metabolite analysis

Total lipid extracts from cultures or scraped TLC spots were dried under nitrogen, solubilised in methanol:chloroform (4:1 v/v), and separated on a ZORBAX Eclipse XDB-C8 column (3.0 × 50 mm, particle size = 1.8 μm; Agilent, USA) using a binary solvent system of water (Solvent A) and methanol (Solvent B). A linear gradient from 80 to 100 % B was applied between 0 and 25 min. Both solvents were supplemented with 5 mM ammonium acetate. The outlet of the liquid chromatograph was connected to a micrOTOF mass spectrometer (Bruker Daltonik, Bremen, Germany) operating in the positive-ion mode and the data were acquired online in the mass range *m/z* 35–1000. MBE products were detected as ammonium, sodium, and proton adducts in the range of 12–18 min of the chromatography run. For quantification of MBW, a calibration curve was done using purified (*Z*)-octyl 2,4,6,8-tetramethylhexacos-19-enoate as standard and MBE concentration in the samples was calculated by linear regression equation obtained from the calibration curve.

### SDS-PAGE and immunoblot

SDS-PAGE and immunoblot analyses using nitrocellulose membranes were carried out using the standard protocols [48]. For detection of the His-tagged proteins, mouse monoclonal anti-His antibodies (QIAGEN™) were used at a dilution of 1:1000. Anti-mouse IgG-alkaline phosphatase conjugates were used as secondary antibodies at a dilution of 1:3000. His-tagged proteins were visualized by immunoblots using chromogenic detection as described by the manufacturer.

### Nile red staining

10^6^ cells of the induced cultures were washed once with PBS and then fixed 15 min in 200 μL of 3 % paraformaldehyde at room temperature. The cells were washed twice with PBS and finally resuspended in 200 μL of PBS. Five microliters of 250-μg/mL nile red in dimethylsulfoxide were added and incubated 5 min at room temperature, 5 μL were used for fluorescence microscopy. The stained bacteria were viewed at 100× oil immersion in a Nikon Eclipse E800 fluorescence microscope using the excitation filters FITC-HYQ (excitation 460–500 nm, emission 510–560 nm). Images were captured using the Nis elements software.

### Fed-batch cultures

For bioprocess optimization, a 2 L bioreactor BioFLO 110 Fermentor/Bioreactor (New Brunswik Scientific^®^) containing 0.9 L of defined minimal medium consisting of 20.8-g KH_2_PO_4_, 0.5-g yeast extract, 3-g (NH_4_)_2_HPO_4_, 3.25-g K(OH), and 4-g NaH_2_PO_4_ was autoclaved at 121 °C for 60 min. After sterilization, 5- or 20-g glucose, 0.125- or 0.5-g MgSO_4_·7H_2_O, 1.5-mL trace element, 2-mL 10-mM biotin solution, and 1-ml 50-mg mL^−1^ kanamycin solution in 90 mL of deionized water were filter-sterilized and added aseptically to the bioreactor. Trace element solution contained 50-mM FeCl_3_, 20-mM CaCl_2_, 10-mM ZnSO_4_, 10-mM MnCl_2_, 2-mM CoCl_2_, 2-mM CuCl_2_, 2-mM NiCl_2_, 2-mM H_3_BO_3_, 2-mM Na_2_MoO_4,_ and 2-mM Na_2_SeO_3_. Temperature, pH, glucose concentration, dissolved O_2_, and OD_600_ were continuously measured along the process. The pH of the medium was maintained at 7.0 ± 0.1 by the addition of 15 % NH_4_OH. When ammonium concentration reached 200 mM, 15 % NH_4_OH was replaced by 5-M NaOH. When necessary, Antifoam A (Code A5633, Sigma Aldrich^®^) was manually added to the vessel to prevent foam formation. Glucose and ammonium concentrations were measured with enzymatic kits from Wiener Lab^®^, codes 1400101 and 1810050, respectively.

The bioreactor was inoculated with 10 ml of RQ5/pMB07 grown in the defined medium described above at an OD_600_ 0.5. Temperature was maintained at 37 °C with an aeration rate of 3 VVM and an initial agitation rate of 600 rpm. After inoculation, a 6- or 10-h batch phase was carried out until glucose was completely exhausted from the medium. Once the glucose in the initial medium was depleted, a nutrient feed solution consisting of 600 g L^−1^ glucose and 15-g L^−1^ MgSO_4_·7H_2_O was initiated at a constant rate of 9.5-g glucose (L h)^−1^ until the culture reached the target OD_600_ for induction. At this moment, the fermentation temperature was reduced to 22, 25, or 30 °C, and heterologous protein expression and MBE production were induced with the addition of 1-, 0.1-, or 0.05-mM IPTG. Simultaneously, 40- or 20-mM sodium propionate, 2- or 1-mM *n*-octanol, and 0.07-mM oleic acid were also added to the culture. 20 mM sodium propionate, 1-mM *n*-octanol, and 0.07-mM oleic acid were also subsequently added as pulses every 12 h when indicated (see main text). After induction, the nutrient feed rate was adjusted to modulate growth rate and to maintain glucose concentration below 10 g L^−1^ during the production phase of the fermentation. The dissolved oxygen was set at 60 % of air saturation by O_2_/air gas supply combined with an agitation cascade between 600 and 1100 rpm. In all cases, the culture was allowed to continue until a decrease in OD_600_ was detected.

For pilot scale MBE production, a 75-L BIOSTAT D-DCU (Sartorius Stedim Biotech) containing 36 L of the defined medium described above was autoclaved in situ at 121 °C for 60 min. After sterilization, 800-g glucose, 20-g MgSO_4_·7H_2_O, 60-mL trace element, 80-mL 10-mM biotin solution, and 40-mL 50 mg mL^−1^ kanamycin solution in 3.6 L of deionized water were filter-sterilized and added aseptically to the bioreactor. Temperature, pH, glucose concentration, dissolved O_2_, generated CO_2_, and OD_600_ were continuously measured along the process. pH was maintained at 7.0 ± 0.1 as described above for 1-L fermentations. When necessary, Antifoam A (Code A5633, Sigma Aldrich^®^) was automatically added to the vessel. Glucose and ammonium concentrations were measured as described above.

The bioreactor was inoculated with 400 ml of RQ5*/*pMB07 culture grown in the defined medium described above at an OD_600_ 0.5. Temperature was maintained at 37 °C with an aeration rate of 3 VVM and an initial agitation rate of 800 rpm. After inoculation, a 10-h batch phase was carried out until glucose was completely exhausted from medium. Once the glucose in the initial medium was depleted, at OD_600_ 23, a nutrient feed consisting of 600-g L^−1^ glucose and 15-g L^−1^ MgSO_4_·7H_2_O was initiated at a constant rate of 9.5-g glucose (L h)^−1^ until the culture reached OD_600_ 80. At this moment, the fermentation temperature was lowered to 22 °C and heterologous protein expression and MBE production were induced with the addition of 0.1-mM IPTG. Simultaneously, 20-mM sodium propionate, 1-mM *n*-octanol, and 20-mg oleic acid were also added to the culture. After induction, the nutrient feed rate was adjusted to set growth rate at 0.04 h^−1^ and to maintain glucose concentration between 0 and 1-g L^−1^. 20-mM sodium propionate, 1-mM *n*-octanol, and 20-mg oleic acid were added as pulses every 12 h during the production phase of the fermentation. The dissolved oxygen was set at 60 % of air saturation by O_2_/air gas supply combined with an agitation cascade between 600 and 1100 rpm. The culture was allowed to continue for 55 h, time at which an evident decrease in OD_600_ was registered. At this moment, cells were harvested by centrifugation and stored at −80 °C.

### MBE extraction and purification

*Escherichia coli* cells obtained from fed-batch fermentation were harvested and total lipids were extracted according to the Bligh and Dyer method [47]. Total lipid extract was loaded onto pre-packed silica gel (60 Å pore size, 35–75-μm particle size) column formerly equilibrated with two column volumes of hexane. A MBE/FA extract was obtained by elution with two column volumes of a mix of hexane:ethyl acetate (90:1 v/v). This MBE/FA extract contains negligible phospholipid contamination, but still contains traces of FA as observed by the LC–MS analysis. Consequently, it was loaded onto a new pre-packed silica gel (60 Å pore size, 35–75-μm particle size) column formerly equilibrated with two column volumes of hexane and eluted with a mix hexane:ethyl acetate (90:3 v/v). Purified MBE fraction was collected in the third column volume fraction. The purity of this MBE fraction was confirmed by the LC–MS analysis.

### MBE physicochemical characterization

Methyl oleate and tetracosanoate reference standards (99+ %) were from Nu Chek Prep (Waterville, MN, USA). Differential scanning calorimetry (DSC) analyses were conducted with a TA Instruments (New Castle, DE, USA) model Q2000 DSC outfitted with an autosampler, a model RCS 90 cooling system, and a model 5000 PC-based controller/analyzer. Nitrogen at 60 mL min^−1^ was employed as a purge gas to eliminate moisture within the DSC cell. The cell was calibrated based on the melting point of indium (156.60 °C) supplied by the manufacturer. Sample mass was 1.486 mg for MBE, 1.520 for methyl oleate, and 1.357 for methyl tetracosanoate. Samples were hermetically sealed in aluminum pans and analyzed alongside an empty reference pan in the measurement cell. The temperature program was: (1) equilibrate at 40 C; (2) hold isothermally at 40 °C for 10 min; (3) cool at 5 °C/min to −85 °C; (4) hold isothermally at −85 °C for 30 min; and (5) heat at 5 °C/min to 60 °C. Heat flow versus temperature data were recorded during cooling and heating ramps with the results being analyzed for the crystallization onset (cooling) and completion of melt (heating) temperatures (T_O_ and T_COM_). DSC results are mean values from three replicate scans (SD ≤ 0.59).

Pressurized-DSC (P-DSC) scans were performed to analyze the oxidative stability of the MBE sample with an induced heating ramp rate. This analysis was conducted using a TA Instruments model Q10P P-DSC with oxygen purge gas flowing at 100 mL min^−1^. The sample (1.478 mg) was sealed in an aluminum pan with a 0.1-mm hole punched in the top and analyzed alongside an empty reference pan (also with a hole punched in the lid). Once the pans were sealed in the P-DSC cell, the cell was pressurized to 3.5 MPa with oxygen and the flow rate set. The temperature program was: (1) equilibrate at 30 °C and (2) heat at 10 °C min^−1^ to 350 °C. After the analysis, the curve was analyzed to yield the onset temperature of oxidation and peak maximum temperatures of MBE (T_OX_ and T_PK_). P-DSC results are mean values from three replicate scans (SD ≤ 0.59).

The pour point (PP) of the MBE sample was analyzed using a Phase Technology (Richmond, BC, Canada) model PSA-70S automatic analyzer following methods outlined in ASTM standard D 5949 [49]. The PP of MBE reported herein is a mean value from three replicate analyses.

## Additional files

10.1186/s13068-016-0631-x Protein expression in different *E. coli* RQ1 derivatives strains. A. Western blot analysis of RQ1 derivative strain expressing extra copies of *S. coelicolor* PCC complex. (1) RQ1/pMB07 induced with 0.1 mM IPTG. (2) RQ1/pMB07/pMB20 not induced. (3) RQ1/pMB07/pMB20 induced with 0.1 mM IPTG. B. Coomasie stained SDS-PAGE of RQ1/pMB07. (1) RQ1/pMB07 not induced. (2) RQ1/pMB07 induced with 0.1 mM IPTG. C. Western blot analysis of RQ1 derivative strain expressing extra copies of papA5. (1) RQ1/pMB07 induced with 0.1 mM IPTG. (2) RQ1/pMB07/pMB04 not induced. (3) RQ1/pMB07/pMB04 induced with 0.1 mM IPTG and 0.2 % l-Ara. D. Western blot analysis of RQ1 derivative strain expressing extra copies of fadD28. (1) RQ1/pMB07 induced with 0.1 mM IPTG. (2) RQ1/pMB07/pMB05 induced with 0.1 mM IPTG and 0.2 % l-Ara.

10.1186/s13068-016-0631-x MBE production in fed-batch fermentations of *E. coli* RQ5/pMB07 using different carbon sources. *E. coli* RQ5/pMB07 fermentations under cultivation conditions #1 were carried out using glycerol (lane 1) or glucose (lane 2) as carbon sources for both batch and fed-batch phases. After cultivation, cells were harvested and total lipids were extracted. Each lane represents total lipid contained in a sample equivalent to 1 ml of culture of OD600 = 6. FFA: Free Fatty Acids, PL: Phospholipids, MBE: Multimethyl-branched long-chain ester.

## Abbreviations

Ara: arabinose
CDW: cell dry weight
DSC: differential scanning calorimetry
FA: fatty acid
IPTG: isopropyl-β-d-thiogalactopyranoside
LC–MS: liquid chromatography coupled to mass spectrometry
MBE: multimethyl-branched long-chain ester
OD_600_: optical density at 600 nm
P-DSC: pressurized-differential scanning calorimetry
SD: standard deviation
TAG: triglyceride
