# Supplementary figures and images for: High cell density production of multimethyl-branched long-chain esters in Escherichia coli and determination of their physicochemical properties
Source: Biotechnol Biofuels. 2016 Oct 14;9:215. doi: 10.1186/s13068-016-0631-x (PMC5064953; doi:10.1186/s13068-016-0631-x)

**A**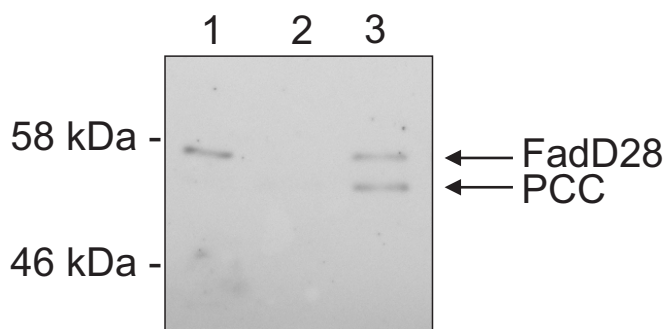**B**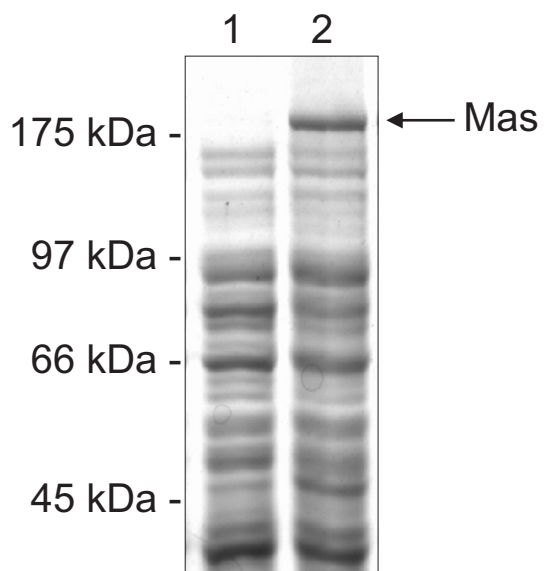**C**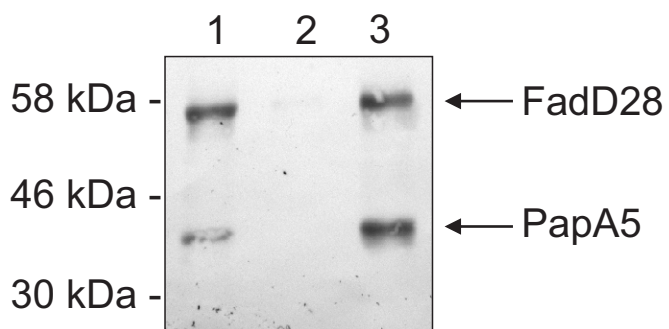**D**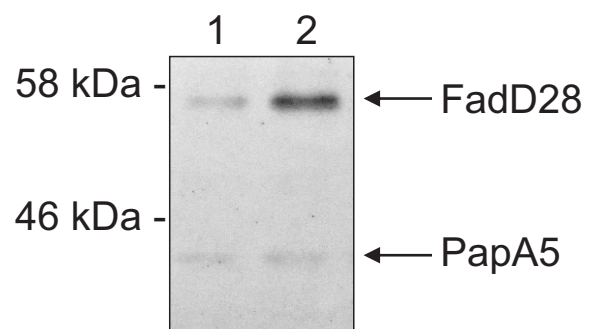

Figure S1

Supplement: Supplementary file 1 — 10.1186/s13068-016-0631-x Protein expression in different E. coli RQ1 derivatives strains. A. Western blot analysis of RQ1 derivative strain expressing extra copies of S. coelicolor PCC complex. (1) RQ1/pMB07 induced with 0.1 mM IPTG. (2) RQ1/pMB07/pMB20 not induced. (3) RQ1/pMB07/pMB20 induced with 0.1 mM IPTG. B. Coomasie stained SDS-PAGE of RQ1/pMB07. (1) RQ1/pMB07 not induced. (2) RQ1/pMB07 induced with 0.1 mM IPTG. C. Western blot analysis of RQ1 derivative strain expressing extra copies of papA5. (1) RQ1/pMB07 induced with 0.1 mM IPTG. (2) RQ1/pMB07/pMB04 not induced. (3) RQ1/pMB07/pMB04 induced with 0.1 mM IPTG and 0.2 % l-Ara. D. Western blot analysis of RQ1 derivative strain expressing extra copies of fadD28. (1) RQ1/pMB07 induced with 0.1 mM IPTG. (2) RQ1/pMB07/pMB05 induced with 0.1 mM IPTG and 0.2 % l-Ara. [file 13068_2016_631_MOESM1_ESM.pdf]

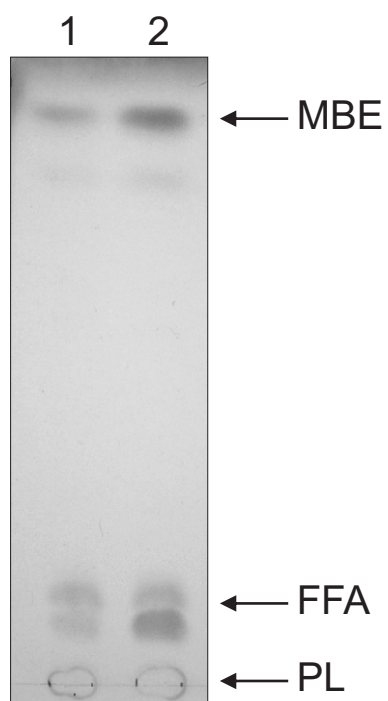

Figure S2

Supplement: Supplementary file 2 — 10.1186/s13068-016-0631-x MBE production in fed-batch fermentations of E. coli RQ5/pMB07 using different carbon sources. E. coli RQ5/pMB07 fermentations under cultivation conditions #1 were carried out using glycerol (lane 1) or glucose (lane 2) as carbon sources for both batch and fed-batch phases. After cultivation, cells were harvested and total lipids were extracted. Each lane represents total lipid contained in a sample equivalent to 1 ml of culture of OD600 = 6. FFA: Free Fatty Acids, PL: Phospholipids, MBE: Multimethyl-branched long-chain ester. [file 13068_2016_631_MOESM2_ESM.pdf]
